# The Influence of Heat Treatment Temperature on Tensile Properties of Metal-Bonded Diamond Composites Fabricated via Selective Laser Melting

**DOI:** 10.3390/ma16206683

**Published:** 2023-10-13

**Authors:** Guangyao Han, Yangli Xu, Jinquan Wei, Guoqin Huang, Tingting Li, Yiqiang He, Zhiping Xie, Zihong Mai, Zeling Yang

**Affiliations:** 1 Institute of Manufacturing Engineering, Huaqiao University, Xiamen 361021, Chinawjqwjq19980428@163.com (J.W.); smarthgq@hqu.edu.cn (G.H.); 22014080024@stu.hqu.edu.cn (Y.H.); 22014080095@stu.hqu.edu.cn (Z.X.); 2 Xiamen Institute of Software Technology, Xiamen 361024, China; 18750928629@163.com; 3 College of Mechanical Engineering and Automation, Huaqiao University, Xiamen 361021, China; 2011114020@stu.hqu.edu.cn (Z.M.); 2011114026@stu.hqu.edu.cn (Z.Y.)

**Keywords:** metal-bonded diamond composites, selective laser melting, heat treatment, tensile properties

## Abstract

Selective Laser Melting (SLM) is an effective technology for fabricating new types of porous metal-bonded diamond tools with complex geometries. However, due to the high cooling rate and internal stresses during SLM fabrication, defects such as high porosities and interface gaps still need to be resolved before it can be considered for use in other applications. The influence of heat treatment temperature on internal characterization, interface microstructures, and tensile properties of AlSi7Mg-bonded diamond composites fabricated by SLM were investigated in this work. From experimental results, the porosities of HT-200, HT-350, and HT-500 specimens were 12.19%, 11.37%, and 11.14%, respectively, showing a slightly lower percentage than that of the No-HT specimen (13.34%). Here, HT represents “Heat Treatment”. For No-HT specimens, an obvious un-bonding area can be seen in the interface between AlSi7Mg and diamond, whereas a relative closer interface can be observed for HT-500 specimens. After heat treatment, the elastic modulus of specimens showed a relative stable value (16.77 ± 2.79~18.23 ± 1.72 GPa), while the value of yield strength decreased from 97.24 ± 4.48 to 44.94 ± 7.06 MPa and the value of elongation increased from 1.98 ± 0.05 to 6.62 ± 0.51%. This difference can be attributed mainly to the disappearance of the solid-solution hardening effect due to the increase of Si content after heat treatment.

## 1. Introduction

With its strong abrasive bonding strength and perfect anti-deformation stiffness, the diamond-grinding wheel is considered to be a high-performance and effective tool for machining materials to products with desired precision. Due to the above-mentioned advantages, metal-bonded diamond-grinding wheels are used to manufacture engines, wafers, and bone implants [1,2]. Due to the diamond’s ability to achieve a high holding force, important bonding materials such as metals, ceramics, and resins are commonly bonded with diamonds to remove unnecessary materials from the workpiece [3]. Traditional grinding tools are mainly prepared by electroplating [4], hot-pressing sintering [5], and brazing technologies [6]. However, due to the increasing requirements of application scenarios of products, the geometry of corresponding diamond-grinding wheels becomes more complex; examples of this complexity include internal cold channels, complex external shapes, and sufficient chip removal space [7]. Therefore, the fabrication technology of a new type of diamond-grinding wheel with complex geometries is of great significance. 

Selective Laser Melting (SLM) is a kind of additive manufacturing technology for fabricating complex and high-performance parts using metals and other composites [8,9], which can reportedly be applied in industrial and medical fields [10]. Meanwhile, SLM technology has proven to be a feasible method of fabricating a porous diamond-grinding wheel [11,12,13]. Tian et al. [14,15,16] fabricated a series of porous diamond-grinding wheels with controllable porosities and types of cellular structures via SLM technology. The grinding properties of SLM-fabricated diamond-grinding wheels were evaluated and compared to the electroplated tools. Experimental results showed that SLM-fabricated porous diamond-grinding wheels have a smaller grinding force and specific grinding energy compared to electroplated tools by a large margin. The exceptional performance of SLM-fabricated diamond tools was also reported by Peng et al. [17]. Duan et al. [18] investigated the forming quality of SLM-fabricated Cu-Sn-Ti/diamond composites with different process parameters. In addition, they found that SLM specimens had better wear resistance compared to the hot pressing sintered specimens. From their works, the performance of new types of diamond tools fabricated by SLM technology showed incomparable advantages in some aspects compared with traditional diamond tools, indicating SLM-fabricated diamond tools could play an important role in manufacturing special industrial products with complex geometry requirements and extreme processing environments.

Although SLM technology is considered an effective and viable method of fabricating high-performance diamond-grinding tools, defects such as high porosities, micro-cracks, and thermal damage pits still exist because of high cooling rates and internal stresses during SLM fabrication. These issues must be overcome when considering their potential for further application [19,20]. Generally, it is difficult to eliminate these defects during the SLM process. Heat treatment is a useful way to eliminate the internal holes and internal stresses of SLM-fabricated materials for improving the mechanical properties of metal alloys, such as AlSi10Mg, Ti6Al4V, and 316L stainless steel [21,22]. The performance-strengthening mechanisms of SLM-fabricated metals are mainly due to the precipitation of the reinforced phase and the change of grain size. However, to the best of the authors’ knowledge, the influence of heat treatment temperature on the properties of SLM-fabricated metal-bonded diamond composites has not yet been studied entirely.

In this regard, the present work aims to investigate whether heat treatments can improve the mechanical properties of SLM-fabricated metal-bonded diamond composites, and which temperature is the appropriate option for their optimal properties. The Computed Tomography (CT) detection, molten pool detection, microscopic interface observation, phase analysis, and tensile test results of SLM-fabricated specimens were carried out. Notably, the effect mechanism of heat treatment temperature on the properties of SLM-fabricated specimens was finally revealed.

## 2. Materials and Methods

### 2.1. SLM Fabrication Equipment and Tensile Specimens Preparation

The SLM fabrication equipment used was the SLM 125^HL^ (SLM Solutions company, Lübeck, Germany), which was equipped with an IPG continuous laser and Melt Pool Monitoring (MPM) system. The entire fabrication process was monitored by the MPM system. MPM is a coaxial inspection tool that allows for the visualization of the heat distribution of the melt pool during the additive manufacturing process on the SLM 125^HL^. The MPM system provides a clear view of the heat distribution of each layer and can be used to evaluate the SLM fabrication parameters. The maximum fabricating size of the SLM equipment is 125 mm × 125 mm × 300 mm, and the fabricating accuracy can reach 140 μm. In order to prevent cracks in the initial layer of the specimens caused by excessive temperature gradients, the powder bed was preheated to 200 °C before fabrication. Meanwhile, it was necessary to introduce the protective gas argon into the fabricating chamber to prevent oxidation of the specimens during the SLM fabrication process. This ensured that the oxygen content was reduced to less than 100 ppm.

**Figure 1:**
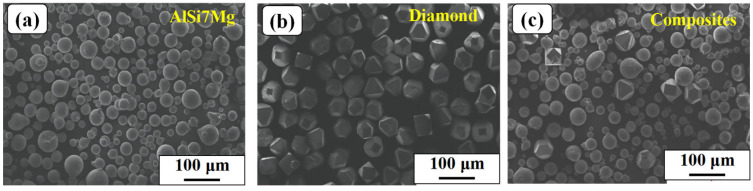
The SEM images of (**a**) gas-atomized AlSi7Mg powder, (**b**) commercial diamond powder and (**c**) mixed metal-bonded diamond composites.

**Figure 2:**
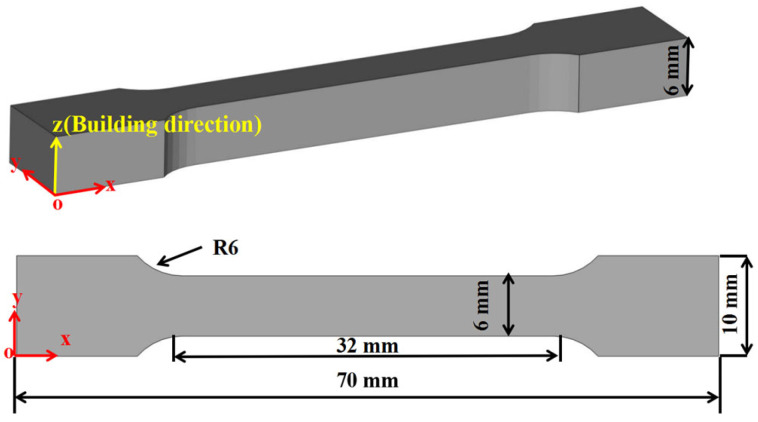
The dimensions of tensile test specimens according to ASTM E8 standard.

As shown in Figure 1a,b, gas-atomized AlSi7Mg alloy powder (Hunan Aoke New Material Technology company, Changzhou, China) with a particle size range of 15–53 μm and a commercial diamond (CR GEMS company, Shanghai, China) with a particle size range of 35–45 μm were used as raw materials for the preparation of metal-bonded diamond composites. The volume concentration of AlSi7Mg and diamond was set at 85% and 15%, respectively. These two powders were placed into the planetary mixer in the specified proportion and mixed for 10 h to obtain the composite powder, as shown in Figure 1c. In order to prevent powder jamming during the SLM process, it was necessary to dry the powder in a vacuum drying box (90 °C + 10 h) before fabricating. The tensile specimens were designed with the shape of a dog-bone based on the ASTM E8 standard [23] shown in Figure 2. The SLM fabrication parameters of metal-bonded diamond composite specimens were set as laser power of 300 W, spot diameter of 80 μm, layer thickness of 40 μm, scanning speed of 3000 mm/s, and hatching space of 120 μm. After SLM fabrication, all specimens were cut from the Al substrate using Wire Electrical Discharge Machining (Jiangsu Chuangwei CNC Machine Tool company, Taizhou, China), and sand blasted for cleaning powder attached to the surface of specimens. Subsequently, the powder adhering to the surface of the specimen was removed using ultrasonic cleaning. All specimens were completely soaked in industrial alcohol during the ultrasonic cleaning process.

### 2.2. Heat Treatment Process

**Figure 3:**
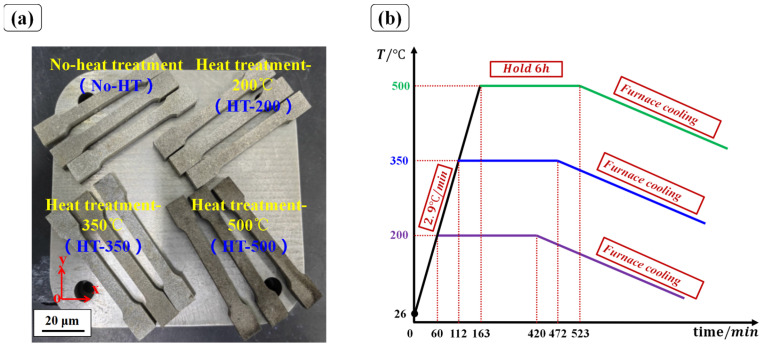
(**a**) Four group specimens with different heat treatment temperatures, (**b**) The heat treatment method of SLM-fabricated tensile specimens.

As shown in Figure 3a, twelve tensile specimens were fabricated and divided into four groups for different heat treatment processes, which were labeled No-HT, HT-200, HT-350, and HT-500, respectively. The first group served as the control group without heat treatment, and the target heat treatment temperatures of the other three groups were 200 °C, 350 °C, and 500 °C, respectively. Figure 3b displays the heat treatment method of SLM-fabricated tensile specimens, that is, three specimens in one group were heated from room temperature to the target heat treatment temperature with a heating speed of 2.9 ℃/min. After that, all specimens were continuously heated for 6 h in an annealing furnace at the target heat treatment temperature. Finally, all specimens were cooled to room temperature and removed from the annealing furnace after 24 h. In this process, the annealed environment of specimens maintained a vacuum of −0.08 MPa via the continuous injection of argon gas with a speed of 10 m/s.

### 2.3. Mechanical Testing and Microstructural Analysis

After heat treatment, one specimen from each group was selected to carry out internal characterization and density measurement using X-ray computed tomography (CT) with a Nikon XT H225 (Nikon company, Tokyo, Japan). Before the tensile test, the surface morphology and interface component Energy Dispersive Spectrometer (EDS) detection between AlSi7Mg and diamond were acquired by the Phenom ProX scanning electron microscope (Phenom-Word BV company, Eindhoven, The Netherlands) under 30 kV. Furthermore, to study the phases of specimens, X-ray diffraction (XRD) was performed on an XRD-7000s (Shimadzu company, Kyoto, Japan) with a Cu tube at 40 kV and 30 mA.

A uni-axial tensile test was carried out using the ProLine-Z100 electronic universal testing machine using ZWICK ROELL equipment (ZwickRoell company, Ulm, Germany) at room temperature. According to the ASTM E8 standard, three specimens of each group were tested, and their stress–strain curves were plotted based on the data from the tensile test. In addition, the mechanical properties including elastic modulus, tensile strength, and elongation were calculated from these stress–strain curves.

## 3. Results and Discussion

### 3.1. Internal Characterization and Volume Ratio Measurement of Specimens

**Figure 4:**
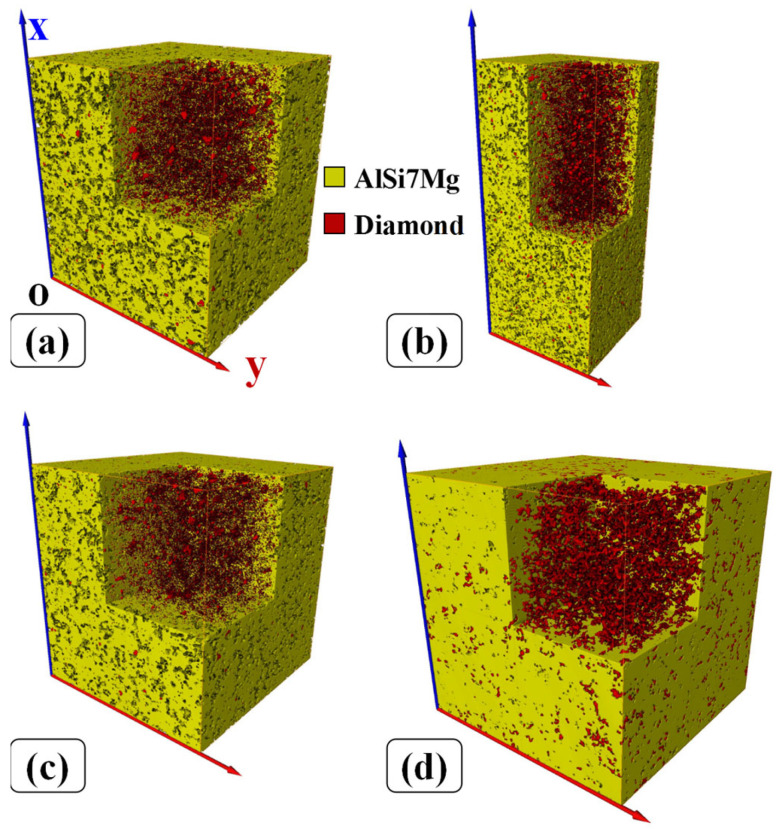
X-ray CT data analysis of different specimens: (**a**) No-HT, (**b**) HT-200, (**c**) HT-350, (**d**) HT-500.

Figure 4 displays the X-ray CT reconstructed models of specimens with different heat treatment processes. Here, only one cuboid region of the specimens was selected to show the 3D spatial distribution of AlSi7Mg and diamond. It can be seen that AlSi7Mg particles are molten absolutely while the diamond still maintains its particle morphology. As expected, diamond particles are evenly distributed on the surface and inside specimens and encased in AlSi7Mg alloy. Table 1 shows the volume ratio of specimens with different heat treatment processes calculated from X-ray CT scanning data. The volume ratio of prepared composite powder is approximately 17.65%; however, this value is slightly lower than those of SLM-fabricated specimens with different heat treatments. This phenomenon may be the result of the difference in melting and solidification mechanisms for AlSi7Mg and diamond. During the SLM process, laser irradiates the surface of the AlSi7Mg particle, and the surface is melted and vaporized into plasma. Under the blow of impulse pressure, the melted AlSi7Mg is compressed to cause some liquid material to leave the molten pool and form the spatter. After cooling, the spatter becomes solid slag and leaves the surface of the specimens [24]. Therefore, the volume fraction of AlSi7Mg in specimens decreases gradually in layer-by-layer fabrication. However, diamonds do not melt by laser irradiation and most of them are covered by a solidified AlSi7Mg alloy. This is the main reason for the change of volume ratio for specimens after SLM fabrication. In addition, it is obvious that the volume ratio of SLM-fabricated specimens after heat treatments seems to be consistent, proving again that the change in volume ratio of specimens should result from the SLM process.

### 3.2. Porosity Characterization and Measurement of Specimens

**Figure 5:**
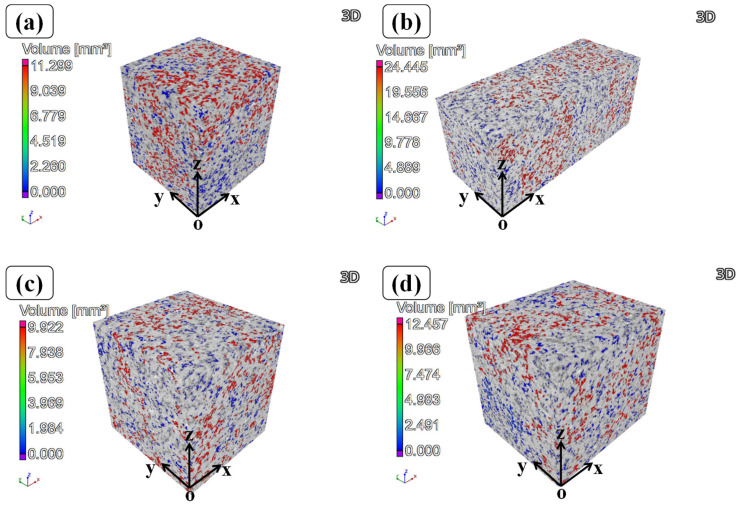
Hole distribution map of specimens with different viewing angles: (**a**) No-HT, (**b**) HT-200, (**c**) HT-350, (**d**) HT-500.

Unlike SLM-fabricated metal material [8], some distinct holes (grayish black region) distributed on the surface of specimens can be seen in Figure 4. In order to further investigate the porosities characterization of specimens, the hole distribution regions are marked in different observation angles, as shown in Figure 5. The three views of specimens after different heat treatments are shown in Appendix A.

**Figure 6:**
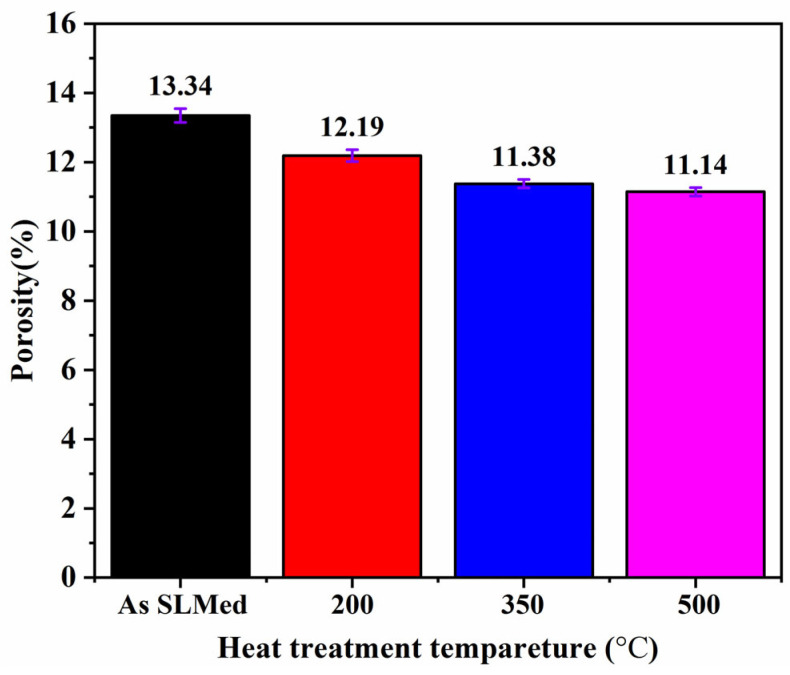
Comparison of porosities of different specimens.

As shown Figure 5, the blue regions represent some independent small holes with the size of 0.5~1 mm^3^ while the red regions represent relatively big holes connected by many holes with the size of 9~10 mm^3^. From these results, either SLM-fabricated or heat-treated specimens display many obvious holes on the surface of and inside the specimens. In order to decrease the porosities, the heat treatment method was adapted to deal with SLM-fabricated specimens. As shown in Figure 6, the porosities of HT-(200~500) specimens are 12.19%, 11.37%, and 11.14%, respectively, showing slightly lower than that of No-HT specimens. Similar results were reported by Arfan Majeed et al. [25]. From their works, the porosities of the heat-treated AlSi10Mg bulk specimens (6 mm × 8 mm × 10 mm) decreased from 0.56% to 0.37%, showing a lower value than the no-heat-treated specimens. The reason for these results is the fine grain structure and strong bonding between particles after heat treatment. Contrarily, the porosity-reducing mechanism in this work may be explained by the closer interface bonding between AlSi7Mg and diamond.

**Figure 7:**
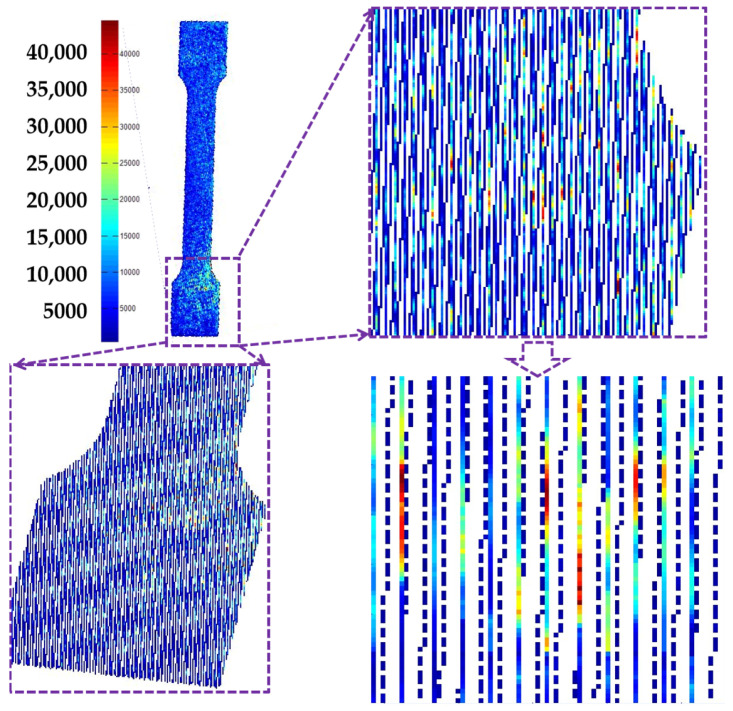
Melt pool monitoring data of specimens during SLM process.

Figure 7 shows the MPM data of the specimen recorded from the SLM fabrication process. The red color represents the high temperature region while the blue color is the low temperature region. An ideal SLM fabricating process should show a consistent temperature field [26]. However, some red and yellow regions appeared in the melt pool monitor data, indicating a large thermal stress existing in the specimens. This may be the main reason for generating porosities in SLM-fabricated specimens. This will be further discussed in the next section.

### 3.3. Interface Observation between AlSi7Mg and Diamond

**Figure 8:**
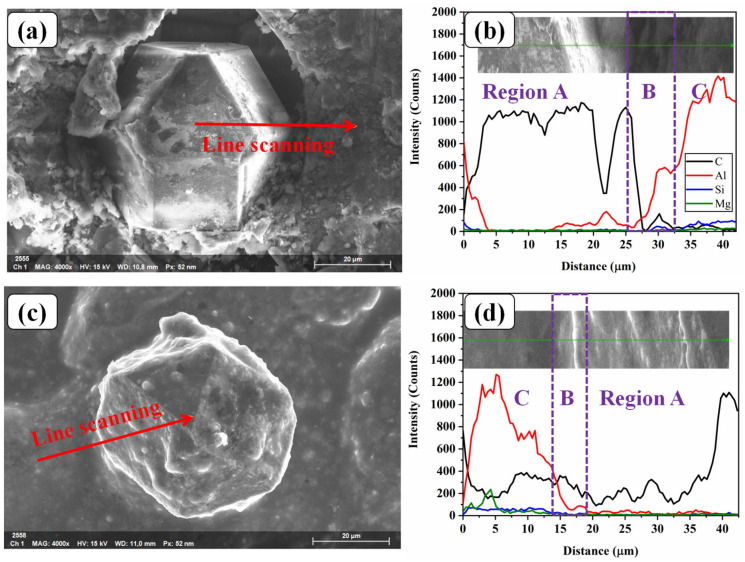
(**a**) SEM image and (**b**) EDS line scan analysis of the AlSi7Mg/diamond interface of No-HT, (**c**) SEM image and (**d**) EDS line scan analysis of HT-500.

Figure 8a,b display the SEM image of AlSi7Mg/diamond interface and EDS line scanning analysis of the No-HT specimen. It can be seen that the diamond was partly bonded with the melted AlSi7Mg alloy, while still maintaining some areas without bonding. From the EDS results, the composition distribution of specimens can be divided into three regions: region A is rich in the C element, representing the diamond particle, while region C is rich in Al, Si, and Mg elements, indicating this area is melted AlSi7Mg alloy. The beginning of region B, marked with the purple wireframe, there is a dramatic decrease in C and Al elements, which indicate a gap between AlSi7Mg and diamond. After heat treatment, the diamond particles can also be seen clearly, and show more apparent bonding with AlSi7Mg in the HT-500 specimens, as shown in Figure 8c,d. The content of the Al element decreases gradually and the content of the C element maintains a stable value in region B. This indicated the interface of AlSi7Mg/diamond may exist in a C-Al compound because diamond would be corroded by melted AlSi7Mg at 800 °C, and Al_4_C_3_ will be produced, which can provide a high binding force between AlSi7Mg and diamond [12]. Thus, these results suggest that heat treatment may enhance the mechanical properties of the composite.

### 3.4. Tensile Test of Specimens

#### 3.4.1. Tensile Properties

**Figure 9:**
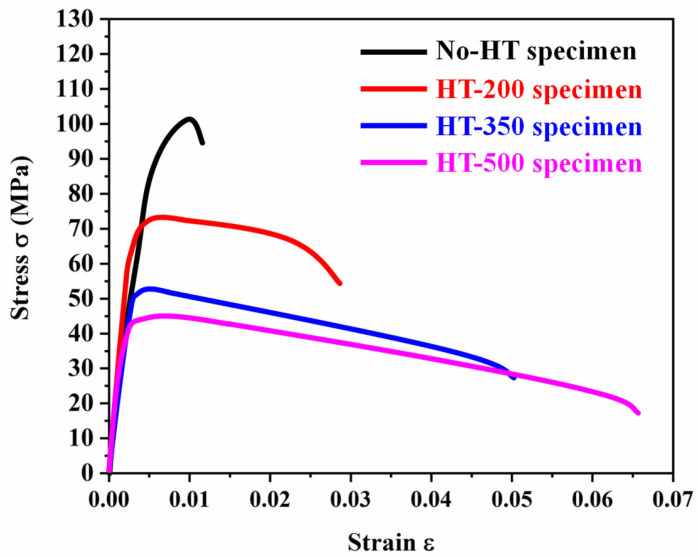
The stress–strain curves of different AlSi7Mg-bonded diamond composites specimens under tensile test.

Figure 9 plots the tensile stress–strain curves of AlSi7Mg-bonded diamond composite specimens with different heat treatment processes. To better present the experimental results, only one curve from each group is shown in the figure. Table 2 summarizes their tensile properties calculated from these curves, compared with some previous works about the tensile properties of heat-treated SLM-fabricated pure AlSi7Mg [21,27]. The specimens of No-HT exhibited an elastic modulus (*E*), yield strength (*σ_y_*_0.2_), and elongation (*El.*) of 17.20 ± 2.78 GPa, 97.24 ± 4.48 MPa and 1.98 ± 0.05%, respectively. As expected, these results were less than the SLM-fabricated pure AlSi7Mg specimens, whose values of *σ_y_*_0.2_ were 299.23 and 236.0 MPa, and *El.* was 14.36% and 15.30%. This indicates that the addition of diamond will weaken the properties of matrix materials due to the increase in porosities. When the heat treatment temperature rose from 200 to 500 °C, the *E* of specimens showed a relatively stable value, whereas the value of *σ_y_*_0.2_ decreased and the value of *El.* increased. The value of *σ_y_*_0.2_ of HT-200 was 73.16 ± 0.47 MPa, and decreased to 44.94 ± 7.06 MPa for HT-500. In contrast, the value of *El.* increased from 3.31 ± 0.07% for HT-200 to 6.62 ± 0.51% for HT-500. A similar tendency in yield strength and elongation for SLM-fabricated pure AlSi7Mg specimens was reported by Tianchun Zou and Woo Jin Hwang’s work [21,27]. From their experimental analysis, the change in tensile properties between heat-treated and SLM-fabricated AlSi7Mg was due to the Si content increase of heat-treated specimens promoting a solid-solution hardening effect, resulting in lower strength and higher elongation of heat-treated specimens than those of SLM-fabricated specimens. This may be an important factor to explain the properties changing of SLM-fabricated AlSi7Mg-bonded diamond specimens after heat treatment.

#### 3.4.2. Fracture Morphology of Specimens

**Figure 10:**
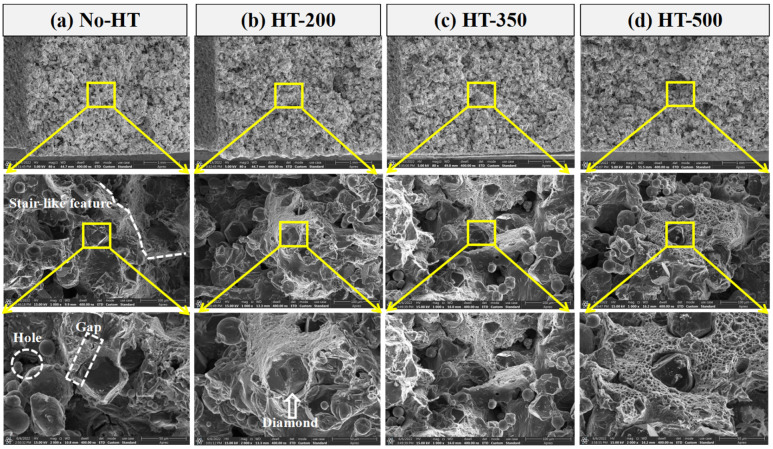
Fracture morphology of different specimens: (**a**) No-HT, (**b**) HT-200, (**c**) HT-350, (**d**) HT-500.

Figure 10a–d illustrates the typical fracture surfaces of No-HT, HT-200, HT-350, and HT-500, respectively observed by SEM. The distribution of diamond and melted AlSi7Mg can be identified clearly. Additionally, some obvious opened up holes and interface gaps (Figure 10a) can be seen on the fracture surface of No-HT. The existence of holes and gaps in composites also acted as crack initiation sites. In addition, there were regular stair-like features observed on the fracture surface, indicating a typical brittle fracture mechanism.

However, the fracture surface of HT-200 began to show a network of dimples in the AlSi7Mg region, indicating a mixed mode of brittle and ductile failure mechanisms. When the heat treatment temperature increased, numerous obvious network dimples can be seen clearly, and diamonds were bonded closely. This phenomenon corresponded to the SEM observations in Figure 8. Specifically, the fracture surface of HT-500 exhibited pure and deep network dimples and their diameter was about 4–5 μm, showing a high ductile fracture mechanism. Therefore, the elongation of specimens was enhanced after heat treatment under suitable conditions.

#### 3.4.3. Property-Changing Mechanism of Specimens

**Figure 11:**
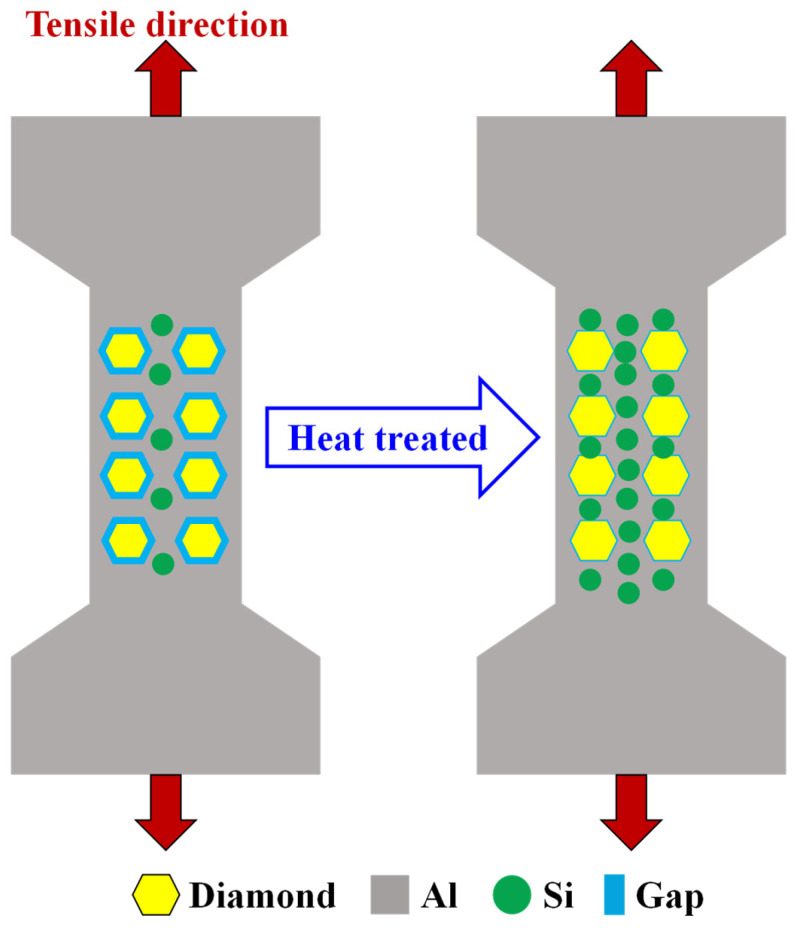
Property-changing mechanism of the SLM-fabricated specimens after heat treatment.

**Figure 12:**
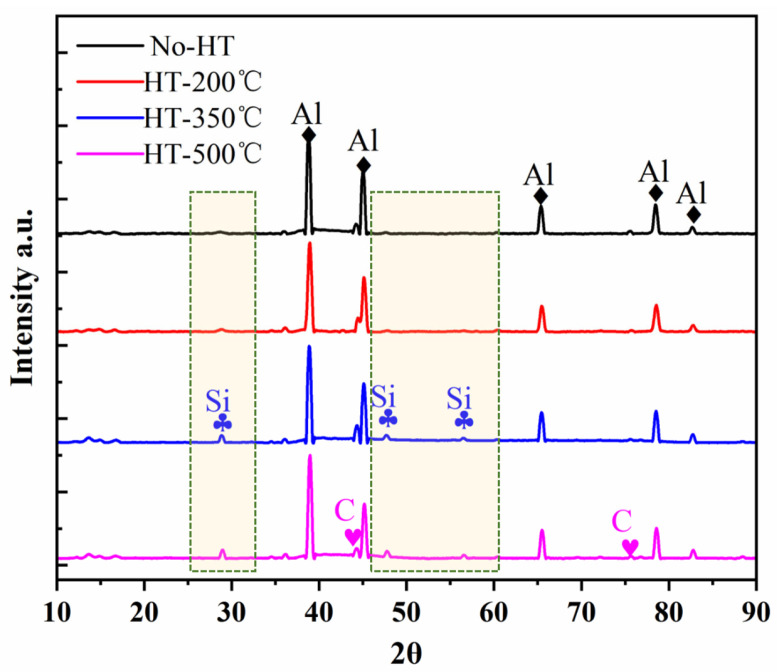
XRD results of the SLM-fabricated specimens before and after heat treatment.

The difference in the tensile properties between the SLM-fabricated and heat-treated AlSi7Mg-bonded diamond composites can be attributed to two factors, as shown in Figure 11. On one hand, the content increase of Si phases in heat-treated specimens will generate the disappearance of a solid-solution hardening effect. This can be proved by XRD and EDS analysis results of the Al and Si element fraction. As shown in Figure 12, it was found that the AlSi7Mg-bonded diamond composites mainly consisted of the C, Al, and Si phases. Among them, the C phase is derived from diamond, while the Al and Si phases are mainly derived from the AlSi7Mg alloy. Interestingly, for No-HT specimens, the intensity peak of Si phases dissolved in the AlSi7Mg matrix material was very low due to the rapid cooling of the SLM process. With the increase of heat treatment temperature from 200 °C to 500 °C, the phase fraction of the Si phase tended to increase because the dissolved Si precipitated, corresponding to the intensity increase of the Si peak in the green box of Figure 12. Furthermore, as shown in Table 3, when the heat treatment temperature increased, the fraction of the Si element increased from 2.32 to 10.27 for HT-500. For the AlSi7Mg matrix material, the increase of the Si element greatly weakened the solid-solution hardening effect, resulting in lower strength and higher elongation for the composites [21]. This is the main reason for property changes of the specimens. On the other hand, as shown in Figure 8, because of the reduction of porosities, the stronger interface bonding between AlSi7Mg and diamond after heat treatment brought higher tensile strength and plasticity of composites. However, the weakening of the solid-solution hardening effect is more dominant than the interface strength due to the reduction of porosities in specimens. Therefore, after heat treatment, the yield strength of AlSi7Mg-bonded diamond composites decreased, whereas the elongation tended to increase.

### 3.5. Evaluations and Further Works of SLM Metal-Bonded Diamond Composites

Considering the fast application of SLM-fabricated AlSi-matrix diamond tools, heat treatment is a useful way to relieve thermal residual stress and promote the interface strength between AlSi-matrix and diamond. A large increase in the elongation of heat-treated specimens can be inferred from this work, indicating a high service life for SLM-fabricated AlSi-matrix diamond tools. Unfortunately, heat treatment also brings a significant reduction in strength of specimens. This is not viable for some application scenarios, such as those requiring compression and shear resistance. Therefore, a suitable heat treatment should be adapted to consider the balance of strength and durability of SLM-fabricated tools.

Additionally, some significant works still need to be explored in the future: (1) Realizing the strong metallurgical combination between metal-bonded materials and diamond by redesigning the structure of diamond powder; (2) Further optimizing the SLM fabricating process for reducing the porosities of specimens; (3) Exploring the appropriate heat treatment process or selecting other metal-bonded materials so that the SLM-fabricated specimens still maintain a high elongation while maintaining a high strength.

## 4. Conclusions

In this work, heat treatment proved to be an effective way to decrease the porosities and increase the elongation of SLM-fabricated specimens. Meanwhile, an obvious strength reduction of specimens can be seen after heat treatment. The conclusions are listed as follows:

1. The volume ratio of prepared composite powder is about 17.65%, showing a slightly lower value than those of SLM-fabricated specimens before or after heat treatment, which had values from 19.04 to 20.90%. This phenomenon may be the result of the melted AlSi7Mg being compressed and causing some liquid material to leave the molten pool and form the spatter. After cooling, the spatter becomes solid slag and leaves the surface of specimens, which were slightly lower than that of No-HT specimens (13.34%).
2. Due to the high cooling rate and thermal stress during the SLM process, the diamond was partly bonded with the melted AlSi7Mg alloy for No-HT specimens. Furthermore, an obvious interface gap between AlSi7Mg and diamond can be observed. After heat treatment, a relatively closer interface and C-Al compound layer can be observed from SEM image and EDS detection.
3. After heat treatment, the elastic modulus of specimens showed a relatively stable value (16.77 ± 2.79~18.23 ± 1.72 GPa), while the value of the yield strength decreased from 97.24 ± 4.48 to 44.94 ± 7.06 MPa, and the value of the elongation increased from 1.98 ± 0.05 to 6.62 ± 0.51%. There were regular stair-like features observed on the fractured surface of No-HT specimens, indicating a typical brittle fracture mechanism. By contrast, the fracture surface of HT-500 exhibits pure and deep network dimples, and their diameter is about 4–5 μm, showing a high ductile fracture mechanism.
4. The difference in the tensile properties between the SLM-fabricated and heat-treated AlSi7Mg-bonded diamond composites can be attributed to porosities decreasing and the disappearance of the solid-solution hardening effect due to the increase in Si content after heat treatment.

## Figures and Tables

**Table 1:** The volume ratio of specimens with different heat treatment processes calculated from X-ray CT scanning data.

Specimens	Volume of Diamond	Volume of AlSi7Mg	Volume Ratio
Composite powder	0.15	0.85	17.65%
No-HT	0.12	0.63	19.04%
HT-200	0.14	0.67	20.90%
HT-350	0.15	0.74	20.27%
HT-500	0.15	0.73	20.55%

**Table 2:** Comparison of tensile properties of AlSi7Mg-bonded diamond composite specimens with pure AlSi7Mg.

Specimens	*E* (GPa)	*σ_y_*_0.2_ (MPa)	*El.* (%)
No-HT	17.20 ± 2.78	97.24 ± 4.48	1.98 ± 0.05
HT-200	18.23 ± 1.72	73.16 ± 0.47	3.31 ± 0.07
HT-350	16.77 ± 2.79	56.97 ± 3.01	5.33 ± 0.41
HT-500	17.93 ± 0.92	44.94 ± 7.06	6.62 ± 0.51
SLMed AlSi7Mg (No-HT) [27]	-	299.23	14.36
SLMed AlSi7Mg (HT-350) [27]	-	210.35	30.83
SLMed AlSi7Mg (No-HT) [21]	-	236.0 ± 0	15.3 ± 0.6
SLMed AlSi7Mg (HT-280) [21]	-	162.0 ± 1.7	17.0 ± 1.7

**Table 3:** EDS analysis results of the Al and Si element fraction (at%).

Specimens	Al (at%)	Si (at%)
No-HT	97.68	2.32
HT-200	95.44	4.56
HT-350	91.35	8.65
HT-500	89.73	10.27

**Figure A1:**
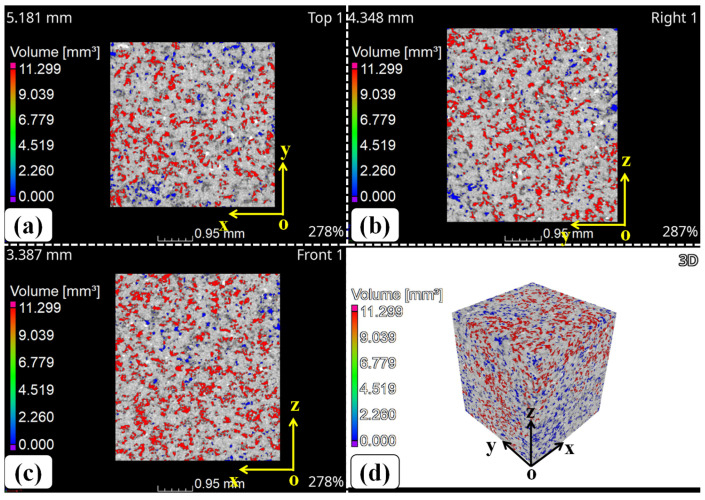

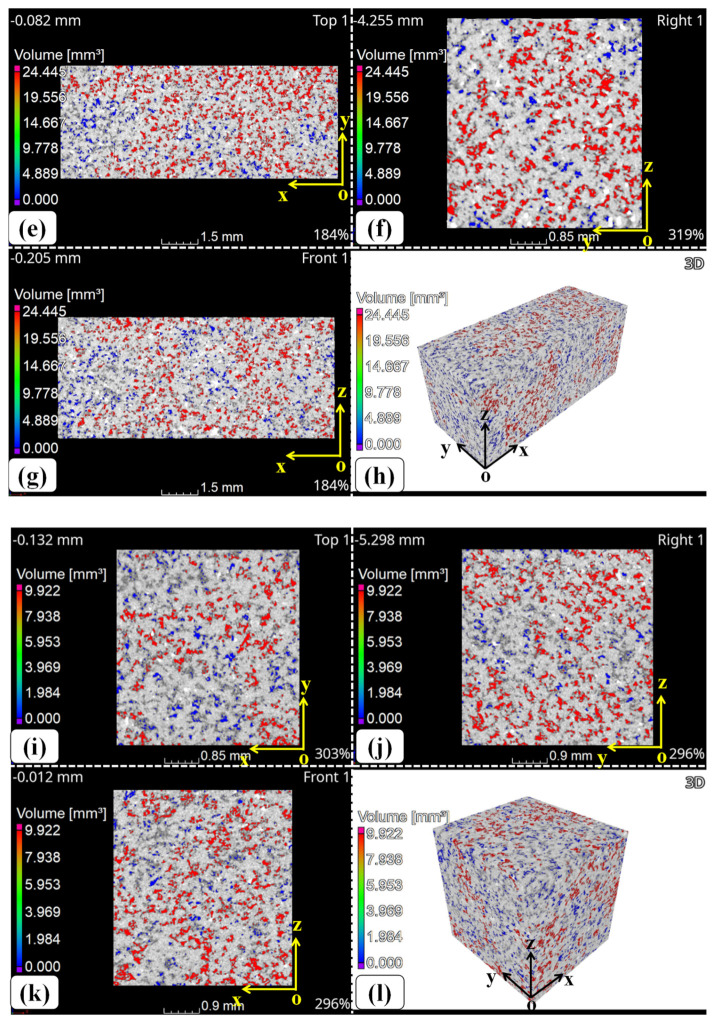

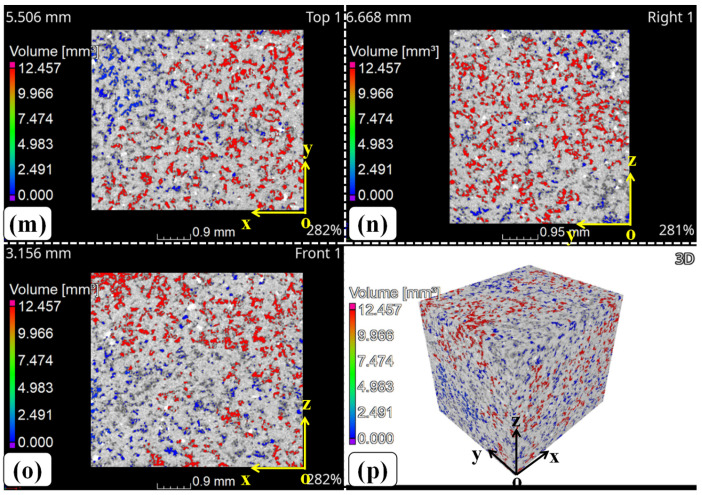
Hole distribution map of specimens with different viewing angles: (**a**–**d**) No-HT, (**e**–**h**) HT-200, (**i**–**l**) HT-350, (**m**–**p**) HT-500.

## Data Availability

Not applicable.
